# Enuresis in Childhood

**Published:** 1902-12-27

**Authors:** Arthur Maude


### Hospital Clinics and Medical Progress.

### ENURESIS IN CHILDHOOD.

By Arthur Maude.

Incontinence of urine in childhood and youth is a common and distressing condition, the causation of which is, however, very obscure, while the general information on the subject is usually ill-arranged and indefinite. Unconscious evacuation of the bladder may occasionally be a continuance of the practice of early infancy, but it more usually manifests itself from the third to the fourth year. It is probably more common in boys than in girls at an early age, but after puberty the habit is more frequently preserved in the female sex. Dr. Eustace Smith's [1] statement that "it is as often seen amongst the strong and robust children as amongst the thin and delicate," is one to which we unhesitatingly demur. Obstinate enuresis persisting into later life is much more common in girls than in young men.

When an infant has acquired control of its bladder, and, later in life the control ceases, we recognise the condition at once as incontinence, and so do the attendants of the child. But if vesical control has never been acquired it may seem difficult to determine where a child has "incontinence of urine." It is often quite possible, and the attempt should begin during the first weeks of life, to teach infants control over their bladders before the end of the first year; usually, however, control during waking hours is not acquired until the course of the second year. If, therefore, during its third year the child has not gained control during waking hours, incontinence may be defined to exist.

We may recall briefly certain structural malformations in anatomy which may produce a continual inability to hold the contents of the bladder. Such conditions are congenital and are very rare, including abnormal opening of the bladder into the vagina, extroversion of bladder, persistence of the urachus, in which case urination occurs through the umbilicus, malposition of the termination of the ureter. In considering the causation of any given case these must be eliminated. Next we must eliminate the possibility of the existence of any disease, degeneracy or non-development of the central nervous system; spinal myelitis, spinal or cerebral meningitis, paralysis of central origin, injuries to the spinal cord, idiocy, true epilepsy, (nocturnal petit-mal), or simple inherited "degeneracy," of which enuresis is a well-recognised "stigma."

The importance and frequency of reflex irritations in the genito-urinary organs or in remote parts of the body have been greatly overestimated, though the existence of such irritations must be borne in mind and never overlooked. There may be phymosis, with a long unclean, perhaps adherent, prepuce; balanitis, perhaps causing narrowing of the urethral orifice. In girls vaginitis has been called in as a cause, and even adhesions of the glans of the clitoris: thread worms in both sexes are claimed as frequent excitants. Further afield, any peripheral irritation from incipient hip disease to adenoids of the naso-pharynx have filed their claim.

It has been long recognised that certain conditions of the urine, by direct irritation of the bladder, may produce incontinence in the child, just as they produce intolerance of the bladder in the adult. One is pyelitis, the result of tuberculous infection of the ureteral tract or of renal calculus—either comparatively rare—the other is a redundancy of uric acid, which is very common. In the adult cystitis is a frequent cause of cystic irritation, and the causes of the cystitis are many, but the writer is convinced that a mild infective cystitis is by no means an infrequent condition in small boys with long uncleanly foreskins. If careful investigation of the urine is made in all cases of phymosis, balanitis and adherent prepuce, in which incontinence is present, the evidence of a mild infection of the bladder will seldom be found lacking.

In treating a case of enuresis the first step is to eliminate any source of external irritation, to remove an elongated or inflamed prepuce, to break down preputial adhesions, to get rid of ascarides if present. The child should be made to empty the bladder on going to bed and again later when the nurse or mother retires. If a high degree of acidity due to uric acid be present, and only if this is known to exist, a diet including little meat and no meat broths, beef-tea, or soups should be adopted, with a very small allowance of sugar, whether raw or in jams. The old plan of restricting the child's drink is thoroughly bad, it merely produces a highly concentrated urine, of which the vesical mucous membrane is very intolerant. In cases where the urine is found of a high specific gravity the child should be encouraged to drink freely towards the end of the day. It is rarely the case that nocturnal enuresis is due to laziness or neglect on the part of the child, and the severe punishments which used to be meted out are illogical and useless. Occasional involuntary passage of urine in the day in children "old enough to know better" is, however, often the result of distension of the bladder from mere neglect to empty it at convenient times.

Drug treatment should always begin with belladonna, and the drug should be pushed so as to produce the ordinary effects, mydriasis, failure of accommodation and dryness of throat. In children of four or five years old we would begin with 25 ♏ or 30 ♏ of the tincture of belladonna three times a day, increasing the dose by 5 ♏ every second or third day; but the physiological effects must be supervised. Children will, however, stand very large doses (Eustace Smith); a better plan still is to give one single large dose at night (Coutts). It has been claimed that when belladonna has failed a combination of belladonna and bromides may be administered at night with very useful effect. Cantharides, which is assumed to produce contraction of the sphincter vesicæ by direct irritation of the mucous membrane, has been at times much vaunted; but it seems occasionally to do more harm than good.

Dr. Coutts [2] places large reliance on lycopodium, a drug the merits of which have not yet been sufficiently proved in enuresis. Twenty minims of the tincture three times a day to a small child, increasing the dose gradually to a drachm. He knows of no poisonous effects produced by the drug, and we have not been able to trace records of any since Dr. Coutts' paper.

In girls and young women very good results have been lately reported to have followed the application of strong solutions of nitrate of silver to the urethra: a solution of 40 to 60 grains to the ounce is to be carried on a small probe covered with cotton-wool, half-way up the urethra, allowing the solution to remain in contact for a minute and a half. Mr. Parnell,[3] who advocates this plan warmly in all obstinate cases, advises the dilation of the urethra by a small double-bladed wire speculum before the passage of the probe. No oily lubricant must be used upon the speculum, as the urethral mucous membrane must remain unprotected, but the vulva must be fully protected by some ointment. No anæsthetic should be used, nor even cocaine if not absolutely necessary. The patient remains in bed for 24 hours, and the application is repeated after an interval of three days, and again in a week. It is difficult to explain the success of the procedure; it may be due to the mental effect, to the reflex irritation of the sphincter muscle, or (more probably) to the slight swelling of the urethral mucous membrane which affords a sufficient, though slight, obstacle to the flow of urine. In the writer's hands this method has been efficacious in several very old-standing and obstinate subjects, though it has failed as often as it has succeeded.

[1] Eustace Smith. Disease in Children, 1884, p. 748. [2] Treatment, Jan. 1898. [3] Brit. Med. Journ., 1892, I., p. 72.

